# Correction: Perceived COVID-19 susceptibility and preventive behaviors: moderating effects of social support in Italy and South Korea

**DOI:** 10.1186/s12889-023-15150-8

**Published:** 2023-02-06

**Authors:** Soontae An, Peter J. Schulz, Hannah Kang

**Affiliations:** 1 Division of Communication and Media, Ewha Womans University, Seoul, 03760 South Korea; 2 Faculty of Communication, Culture and Society, Università della Svizzera italiana, Lugano, Switzerland; 3 Department of Politics and Communication Studies, Hannam University, Daejeon, 34430 South Korea

**Correction: BMC Public Health 23, 13 (2022)**

**https://doi.org/10.1186/s12889-022-14866-3**

The original publication of this article [1] was missing the following funding acknowledgement: This work was supported by the National Research Foundation of Korea (NRF-2020S1A5C2A 03092919). The original publication has been updated.
